# AML1/ETO and its function as a regulator of gene transcription via epigenetic mechanisms

**DOI:** 10.1038/s41388-021-01952-w

**Published:** 2021-07-30

**Authors:** Kai Rejeski, Jesús Duque-Afonso, Michael Lübbert

**Affiliations:** 1 Department of Hematology, Oncology and Stem Cell Transplantation, University of Freiburg Medical Center, Freiburg, Germany; 2 Department of Hematology and Oncology, University Hospital of the LMU Munich, Munich, Germany; 3 German Cancer Consortium (DKTK) Freiburg Partner Site, German Cancer Research Center (DKFZ), Heidelberg, Germany; 4 Faculty of Medicine, University of Freiburg, Freiburg, Germany

**Keywords:** Acute myeloid leukaemia, Predictive markers

## Abstract

The chromosomal translocation t(8;21) and the resulting oncofusion gene *AML1/ETO* have long served as a prototypical genetic lesion to model and understand leukemogenesis. In this review, we describe the wide-ranging role of AML1/ETO in AML leukemogenesis, with a particular focus on the aberrant epigenetic regulation of gene transcription driven by this AML-defining mutation. We begin by analyzing how structural changes secondary to distinct genomic breakpoints and splice changes, as well as posttranscriptional modifications, influence AML1/ETO protein function. Next, we characterize how AML1/ETO recruits chromatin-modifying enzymes to target genes and how the oncofusion protein alters chromatin marks, transcription factor binding, and gene expression. We explore the specific impact of these global changes in the epigenetic network facilitated by the AML1/ETO oncofusion on cellular processes and leukemic growth. Furthermore, we define the genetic landscape of AML1/ETO-positive AML, presenting the current literature concerning the incidence of cooperating mutations in genes such as *KIT, FLT3*, and *NRAS*. Finally, we outline how alterations in transcriptional regulation patterns create potential vulnerabilities that may be exploited by epigenetically active agents and other therapeutics.

## Introduction

Acute myeloid leukemia (AML) has long served as a prime model for our understanding of the initiation and propagation of cancer [1]. This is a reflection of the diagnostic accessibility of leukemic cells, the long tradition of implementing routine cytogenetics into the diagnostic workup, as well as the low number of mutations that drive the disease process compared to other cancer entities [2]. The last decade has yielded astonishing progress on dissecting the genetic landscape that lies at the root of AML [3]. Critical advances in the understanding of the molecular mechanisms driven by specific genetic lesions have resulted in key therapeutic advances, such as the FDA approval of FLT3- and IDH1/2-directed therapies [4, 5]. Among the different genetic and biological subtypes of AML, the disease entity with the translocation (8;21)(q22;q22) maintains a prominent position. The functional role of the resultant oncofusion protein AML1/ETO (also termed RUNX1/RUNX1T1) has been studied since its discovery in the early 1990s, and yet new aspects of its function continue to emerge. This is especially relevant since—in contrast to the PML/RARα oncofusion found in APL—a specific, biologically driven treatment approach resulting in a high rate of cure, remains elusive for this AML subtype.

The translocation was the first balanced chromosomal translocation ever described in leukemia or any other cancer [6], and AML harboring t(8;21) constitutes one of the most frequent recurring genetic subtypes of AML [3], especially in childhood AML [7]. It serves as a unique example of how one cytogenetic abnormality can define a distinct leukemia entity: t(8;21) leukemia is associated with a distinct morphology (i.e., relatively large blasts with a basophilic cytoplasm, azurophilic granules, and perinuclear clearing, presence of Auer rods), immunophenotype (i.e., frequent aberrant expression of CD19, PAX5, and CD56) and recurrent cooperating mutations including *KIT, FLT3, KRAS*, or *NRAS*, and both *ASXL1* and *ASXL2* [8]. The AML1/ETO fusion represents one of the first fusion genes employed for minimal residual disease monitoring [9]. Together with AML with inversion (16) or translocation (16;16), this “Core-Binding Factor” AML displays significantly better outcomes with standard chemotherapy followed by high-dose cytarabine consolidation than most other AML subtypes. Despite a cure rate of 60% or higher (including allografting in patients that relapsed after standard chemotherapy) in patients 60 years and younger, the relapse rate and outcome are still strinkingly inferior to APL, particularly in elderly patients who are not candidates for standard chemotherapy due to significant comorbidities. The conundrum of a tantalizingly growing understanding of the functional AML1/ETO and the lack of real improvements in outcome (with the exception of continuously improved outcomes in allogeneic stem cell transplantation) continues to drive basic research of t(8;21) leukemia.

In this review, we will focus on recent results highlighting the role of AML1/ETO as an epigenetic modifier, which provides a strong rationale to treat the disease with so-called epigenetically active agents, and we will emphasize recent findings on cooperating oncogenes that can be targeted by kinase inhibitors. Outlining the sequelae of AML1/ETO-mediated epigenetic dysregulation becomes all the more important since it is notoriously difficult to target the function of aberrant transcription factors directly.

### AML1 (RUNX1), ETO (RUNX1T1), and AML1/ETO: structure and functions

**Fig. 1:**
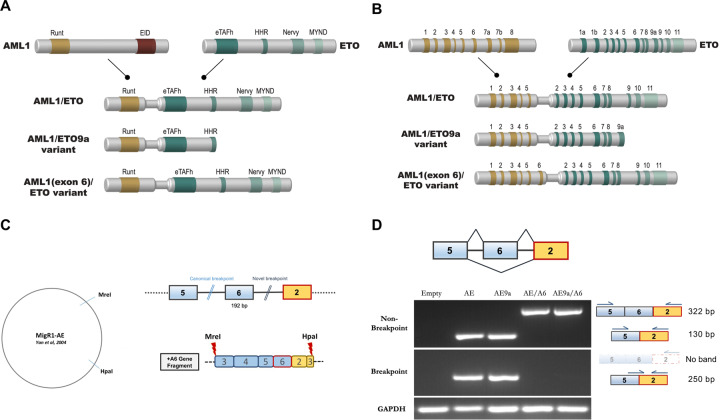
Schematic representation of AML1 (RUNX1), ETO (RUNX1T1), and AML1/ETO—structure and function. **A** The Runt DNA-binding domain of AML1 and almost the whole co-repressor gene ETO are conserved in the fusion gene including its four functional domains termed Nervy Homology domains 1–4 (NHR 1–4): TATA-binding protein-associated factor homology domain (eTAFH = NHR1), the hydrophobic heptad repeat domain (HHR = NHR2), an α-helical domain (Nervy = NHR3), and the myeloid-Nervy-DEAF1 domain (MYND = NHR4). The AML1/ETO 9a variant contains only the NHR1 and NHR2 functional domains, while the AML1(exon 6)/ETO variant contains 64 additional amino acids downstream of the Runt domain with yet unknown functional consequences. **B** mRNA splice variants identified in t(8;21) leukemia include the canonical AML1/ETO, the oncogenic AML1/ETO9a, and other rare variants such as AML1(exon 6)/ETO. **C** The AML1(exon 6)/ETO variant observes a novel breakpoint between AML1 exon 6 and ETO exon 2. Double-stranded synthetic DNA fragments can be utilized to clone novel AML1/ETO splice variants into the retroviral MSCV-IRES-GFP overexpression construct (ref. [21]) by utilizing intrinsic restriction enzyme sites. **D** RT-PCR using exon-specific and exon-junction-spanning primers for the AML1 exon 6 splice event following retroviral transfection of 293T cells with the novel AE/AE6 and AE9a/A6 constructs, as well as the previously published AE and AE9a constructs and a no transfection control (Empty). AE = AML1/ETO, AE9a = AML1/ETO9a variant, AE/A6: AML1(exon 6)/ETO variant, AE9a/A6 = AML1(exon 6)/ETO9a variant. This figure includes original work (see “Acknowledgements”).

The transcription factor *AML1* (*RUNX1*) represents a crucial regulator of physiologic hematopoietic differentiation [10] and is recurrently mutated in a wide variety of hematologic malignancies [11]. Together with other lineage-specifying transcription factors including members of the ETS and GATA family, AML1 coordinates definitive hematopoiesis in a spatial and temporal manner [12]. The full-length protein is comprised of a N-terminal Runt-homology domain responsible for DNA-binding to target gene promoters and nuclear interaction with the common heterodimeric partner Core-Binding Factor β (CBFβ), as well as a C-terminal transactivation domain (TAD) that consists of a number of activating and inhibitory domains, such as the Ets1 interacting domain (EID) (Fig. 1A, B). The EID domain facilitates protein–protein interactions, including with the histone acetyltransferases P300 and CREBBP (CBP). An adjacent inhibitory domain of AML1, located towards the C-terminus of the activation domain, acts to limit the interaction between AML1 and transcription factors such as *ALY* and *YAP* [13, 14].

Endogenous *ETO* (*MTG8, RUNX1T1*) encodes a Zinc-binding protein also named CBFA2T1, a nuclear protein which functions as a transcriptional co-repressor through its close association with transcription factors and by recruiting other corepressors and histone deacetylases. The ETO protein domain structure consists of four highly conserved functional domains termed Nervy Homology domains 1–4 (NHR 1–4). These can be further characterized into the TATA-binding protein-associated factor homology domain (eTAFH = NHR1), which interacts stably with E-Box binding proteins [15], the hydrophobic heptad repeat domain (HHR = NHR2) which is essential for the activity of the AML1/ETO fusion protein [16], an α-helical domain (NHR3), and finally the myeloid-Nervy-DEAF1 domain (MYND = NHR4) (Fig. 1A, B). While ETO on its own lacks DNA-binding capacity, it harbors potent transcriptional repression domains that are preserved as corepressors in the context of the oncogenic fusion protein [17]. Structure–function studies localized AML1/ETO-mediated transcriptional repression to the NHR2–4 region of ETO [18, 19]. In the context of the AML1/ETO fusion protein, ETO is able to interact with a conserved domain of the corepressors NCoR and SMRT via its zinc finger domain, thereby recruiting the histone deacetylase (HDAC) complex in vivo [20]. Deleting the C-terminus of ETO abrogates NCoR binding and HDAC recruitment and impedes the ability of AML1/ETO to inhibit hematopoietic differentiation. The N-terminal 31 amino acids of ETO missing in the AML1/ETO fusion protein are not known to be part of a functionally relevant protein domain.

The t(8;21) translocation generates a canonical genomic breakpoint that lies between *AML1* exon 5 and *ETO* exon 2. The chromatin organization at intron 5 of the *AML1* gene, where most but not all of the sequenced breakpoints have been mapped, predisposes to chromosomal stress via an epigenetic signature that is rich in histone H3 hyperacetylation and characterized by low histone H1 levels [21]. While the simple reciprocal translocation represents the most common source of AML1/ETO fusions, rare variants such as inversions (i.e., inv(8)(q22q24)) and insertions (i.e., ins(21;8) and ins(8;21)) involving the derivative chromosome 8 have been described. Next to t(8;21), more than 50 chromosomal translocations have been attributed to *AML1* [22], illustrating its far-reaching role in tumorigenesis. This includes t(12;21), which generates the *TEL/AML1 (ETV6/RUNX1)* fusion gene product and represents the most common chromosomal translocation in childhood acute lymphoblastic leukemia (ALL). Interestingly, the TEL/AML1 and AML1/ETO fusions can be traced in Guthrie cards in healthy neonates and can be detected prenatally, supporting prenatal initiation and a two-hit model of leukemia inception [23]. Another prominent example is the AML1/MDS/EVI1 (*RUNX1/MECOM)* fusion mediated by the t(3;21), which is recurrently found in patients with therapy-related MDS and AML.

### AML1/ETO splice variants observe differential leukemic potential

Importantly, the resultant full-length AML1/ETO fusion protein (752 amino acids = aa) itself is not sufficient to drive leukemogenesis, but rather provides a crucial first hit. Early conditional knock-in mouse models demonstrated that the full-length fusion requires additional mutagenic events to induce leukemia on its own. While transgenic mice expressing only the full-length AML1/ETO fusion did not develop leukemia, exposure to the DNA alkylating agent ENU (also known as N-ethyl-N-nitrosourea) resulted in the rapid development of a malignant state that mimicked the morphologic cues found in t(8;21) leukemia [24]. In a seminal study, Yan et al. found that one mouse, transplanted with AML1/ETO-transduced bone marrow cells, developed leukemia even in the absence of mutagenic stress [25]. Sequencing of leukemic cells in this mouse revealed a 1-bp insertion that leads to a C-terminally truncated form of AML1/ETO lacking 200 amino acids (552 aa) in a domain critical for the NCoR/SMRT and ETO interaction. A transcriptional isoform harboring an additional exon, termed exon 9a, of *ETO* was discovered to result in a similarly truncated AML1/ETO protein [26]. This alternatively spliced isoform called AML1/ETO9a (572 aa) was recurrently found in a multitude of t(8;21) AML samples [27]. Moreover, co-expression of both the full-length and C-terminally truncated AML1/ETO fusion proteins facilitated a substantially earlier onset of leukemia and blocked myeloid differentiation at an earlier stage [27]. These early studies shed light on how fusion proteins arising from alternatively spliced isoforms secondary to a chromosomal translocation can act in concert to drive the development of cancer.

Sequencing of t(8;21) primary AML samples has revealed a plethora of in-frame and out-of-frame transcript variants arising as a result of alternative splicing. For example, one *ETO* variant containing an additional exon 11a produces a protein with an additional 27 amino acids in-frame instead of the MYND domain at the C-terminal region of the fusion protein. Identified in primary human t(8;21) AML cells, the MYND-less protein variant was associated with the formation of multimers and reduced transcriptional repressor activity [28]. Mannari et al. describe a transcript harboring an alternative exon 6a leading to a protein that only contains the NHR1 domain [29]. As this fusion protein did not exhibit clonogenic potential compared to the leukemogenic AML1/ETO9a fusion, which includes both the NHR1 and 2 domains, the authors conclude that the homo-oligomerization function conferred by the NHR2 domain likely plays a key role in promoting leukemogenesis. In pediatric t(8;21) AML, transcript variants containing multiple in-frame-deletions involving exons 2–5 of *AML1* and exon 2 and 3 of *ETO* were identified, which displayed both activating and repressive effects on AML1-mediated GM-CSF transactivation [30].

While the natural breakpoint observed in t(8;21) leukemia produces an *AML1* exon 5 to *ETO* exon 2 fusion, Solari et al. described a rare novel *AML1/ETO* fusion transcript tightly associated with *BCR/ABL*, wherein the breakpoint lies one intron downstream, resulting in a fusion transcript including *AML1* exon 6 [21]. While the functional consequences of this novel fusion transcript remain to be explored, the association of AML1/ETO and BCR/ABL in cases of therapy-refractory CML is especially intriguing, as these genetic aberrations can coexist together in vivo. As whole-genome sequencing approaches become more wide-spread in diagnosing the genetic landscape of AML [31], more such rare AML1/ETO variants may be identified. Detailed sequencing enables the design of synthetic DNA fragments, which can be utilized to modify established retroviral constructs using AML1/ETO intrinsic restriction enzyme sites. In our laboratory, we have leveraged these new technologies to clone and express the previously described AML1(exon 6)/ETO variant (Fig. 1C, D). These constructs allow variant-specific characterization of AML1/ETO function.

Finally, the presence of spliceosomal mutations in myeloid malignancies has been demonstrated to impact the alternative splicing of the terminal exon of *AML1* [32], and splicing changes related to exon 6 of *AML1* differentially regulate hematopoiesis in mice [33]. Moreover, recent work points toward AML1/ETO itself being a potential regulator of alternative splicing, adding a novel layer of transcriptome organization in t(8;21) leukemia [34].

### AML1/ETO undergoes posttranslational modifications controlling its function

Posttranslational modifications regulate protein–protein interaction and the functional activity of transcription and thus play an important role in oncogenesis [35]. In a seminal work, it was reported that the histone acetyltransferase P300 acetylates *AML1/ETO* at lysine 43 (Lys43), thus enhancing AML1/ETO activating functions and self-renewal activity of hematopoietic progenitor cells. Treatment with P300 inhibitors decreases *AML1/ETO* acetylation, leading to a blockage of AML progression [36]. Furthermore, a recent study demonstrated that AML1/ETO can increase CD48 expression via AML1-ETO/P300-mediated acetylation. CD48, a member of the SLAM family, plays an important role in regulating natural killer (NK) cell-mediated immunosurveillance. By increasing CD48 expression levels, AML1/ETO can inhibit AML immune escape from NK cell recognition and killing [37].

Recently, the interaction between the histone methyltransferase *EZH1* with *AML1/ETO* was shown. *EZH1*, which is part of the Polycomb repressive complex 2 (*PRC2*), methylates Lys43 on the NHR1 domain in *AML1/ETO*, thus enhancing its repressive function on tumor suppressor genes. Hence, loss of Lys43 methylation by point mutation or domain deletion impairs AML1/ETO-repressive activity [38]. These data suggest that *P300* and *EZH1* compete for binding and modification of Lys43 (acetylation and methylation), which confer opposite functions in AML1/ETO-mediated transcriptional regulation. The protein arginine methyltransferase 1 (*PRMT1*) has also been shown to interact directly with AML1/ETO-9a and to methylate the arginine residue at position 142 of the AML1/ETO9a variant. Through this interaction, PRMT1 is recruited to AML1/ETO target promoters and methylates H4R3, which enhances transcriptional activation [39]. Of note, PRMT1 has been shown to interact with the N-terminus of AML1, thereby enhancing its transcriptional activity by inhibiting the interaction with SIN3a [40], suggesting a key role in physiological gene activation of both AML1 and the concomitant AML1/ETO oncofusion protein.

### AML1/ETO recruits multiple chromatin-modifying enzymes to target genes

**Fig. 2:**
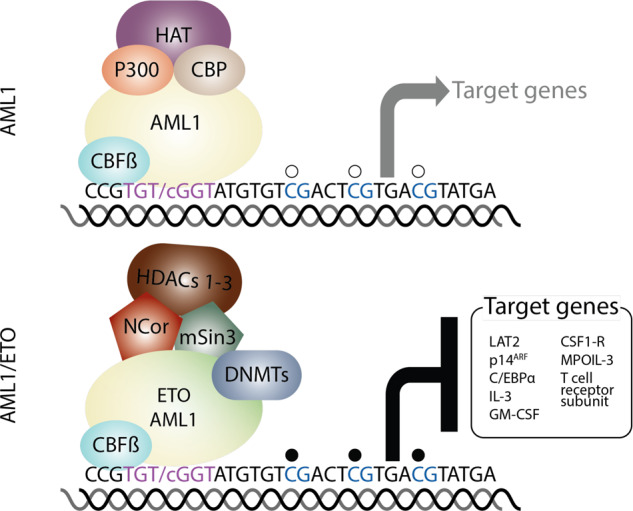
The AML1/ETO oncofusion protein but not wild type AML1/RUNX1 recruits a repressor complex. The hematopoietic transcritption factor AML1/RUNX1 binds the consensus sequence TGTGGT on the promoter of its target genes. DNA binding is stabilized by the interaction with CBFß. AML1 recruits the histone acetyltranferases p300 and CBP. The histone acetyltransferases acetylates lysine residues on the histones of its target genes, which induce an open chromatin and activates gene transcription. However, AML1/ETO interacts with NCOR and mSin3, which recruit class I histone deacetyltransferases (HDACs) 1–3. HDACs1–3 deacetylates the lysine residues of histone tails, which change to a closed chromatin conformation resulting in the repression of transcription of target genes. Some evidence supports that DNA methyltransferases (DNMTs) and the polycomb repressor complex 2 (PRC2) including the H3K27 trimethylase EZH2 are directly or indirectly recruited to AML1/ETO target genes.

A multi-protein complex is recruited by AML1/ETO to target genes, thus epigenetically modifying chromatin and regulating gene transcription. ETO recruits a nuclear co-repressor complex containing HDACs (histone deacetylases) 1–3 via its interaction with NCoR and SIN3A to the promoters of its target genes, acting as a transcriptional repressor (Fig. 2). This repressive effect on transcription is facilitated when the recruited HDACs de-acetylate histones, changing the chromosome structure to a more close conformation on AML1/ETO target promoters [41, 42]. AML1/ETO has been shown to interact—directly or indirectly—with DNMTs, as was demonstrated on the interleukin-3 (IL-3) promoter. At the IL-3 promoter, AML1/ETO is part of a repressive complex containing HDAC1 and DNMT1, whose function can be inhibited with the treatment of the DNA demethylating agent decitabine [43] or with the HDAC inhibitor valproic acid combined with decitabine [44]. A complex constituted by AML1/ETO and DNMT1 was also demonstrated to be physically associated with the *RARβ2* promoter, linking the two major epigenetic changes (histone modifications and DNA methylation) in the molecular pathogenesis of AML1/ETO [45].

Repressive chromatin modifications mediated by AML1/ETO have been described in target genes by several groups. Buchi et al. [46] described the redistribution of H3K27me3 and acetylated H4 by AML1/ETO on the IL-3 promoter, whereas DNMT inhibition reversed silencing marks, particularly H3K27me3, only in AML1/ETO-expressing cells. Chromatin modifications were found on the *LAT2* promoter and introns, a target gene of AML1-chimeric fusion proteins in AML and ALL [47, 48]. In t(8;21) AML*, LAT2* is downregulated as a result of the repressor activity of AML1/ETO. The adaptor molecule is closely regulated during myeloid differentiation [49]. Moreover, LAT2 interferes with differentiation of normal hematopoietic precursor cells, and recent studies highlight the role of LAT2 as a prognostic marker in other leukemia entities such as APL [50, 51]. AML1/ETO induced changes in several histone marks including acetylation of histone H3, H3K9, and H4 as well as di- and trimethylation of H3K9 and trimethylation of H3K27 and H3K4. Interestingly, class I-HDAC inhibitors reversed not only acetylation of H3, H4, and H3K9 but also trimethylation of H3K4, suggesting an interplay of inactivating and activating histone-modifying enzymes on target genes by AML1/ETO or by HDAC inhibitors [52]. Moreover, AML1/ETO physically interacts with the PRC2 component EZH1, recruiting histone methyltransferase activity to its target genes [38]. These data suggest a functional interaction between AML1/ETO and the PRC2, which is also able to recruit DNMTs [53].

AML1/ETO is not only able to act as a repressor but also as an activator of target genes, through its interaction with transcriptional activators (Table 1). AML1/ETO interacts with P300, recruiting the histone acetyltransferase to its target genes. Of note, an increase of histone acetylation was detected in genes activated by AML1/ETO, but not by repressed genes, suggesting recruitment of AML1/ETO-P300 complex to specific genes [36]. Similarly, PRMT1 is recruited by AML1/ETO to its target genes, thus increasing H4R3 methylation on target promoters and activating gene transcription [39]. Therefore, AML1/ETO might behave as an adaptor protein inducing transcriptional stimulation or repression depending on the activated signaling pathways in leukemia cells.

**Table 1:** Selected studies of global epigenomic profiling and transcription factor binding specifically mediated by AML1 and AML1/ETO.

Chromatin marks	Transcription factors	Method	Cell type	Reference
Acetylation				
H3K9ac, H3K14ac, H4panAc	ERG, FLI1, CBFB, HEB, RUNX1, ETO, AML1-ETO, RNAPII	ChIP-Seq	Cell lines, patient t(8;21) AML blasts and normal CD34+ hematopoietic cells	Martens et al. [66]
H3ac	AML1/ETO, AML1, LMO2, Pol II, Pu.1, C/EBPα, HDAC2, P300	ChIP-Seq DNAseI Footprinting	Cell lines, primary cells, normal. CD34+	Ptasinska et al. [58]
H3K9ac	AML1/ETO, AML1, Pol II	ChIP-Seq DNAse I hypersensitivity	Primary cells and cell lines	Ptasinska et al. [67]
H3K27ac	AML1/ETO	ChIP-Seq DNAse I hypersensitivity	Cell lines, primary AML cells, AML1/ETO-transduced iPSCs	Mandoli et al. [70]
H2A.Zac	AML1/ETO, P300	Nuclease accessibility coupled with high-throughput sequencing (NA-seq) and ChIP-seq	Cell lines and primary patient blasts	Saeed et al. [69]
K43ac	AML1/ETO, P300	ChIP-Seq	Cell lines	Wang et al. [36]
H3K9/14ac	AML1/ETO9a, PRMT1	ChIP-qPCR	Cell lines	Shia et al. [39]
H4ac loss	AML1/ETO, SP1	ChIP-chip analysis	Transduced human HSPCs	Maiques-Diaz [118]
Methylation				
H3K4me3, H3K4me1, H3K36me3, H3K27me3, H3K9me3	AML1/ETO	ChIP-seq, DNA hypersensitivity	Cell lines, primary AML cells, AML1/ETO-transduced iPSCs	Mandoli et al. [70]
H3K4me3, H3K27me3	AML1, NCoR, P300	ChIP-Seq	Cell lines	Trombly et al. [72]
H4R3me2a	AML1/ETO9a, PRMT1	ChIP-qPCR	Cell lines	Shia et al. [39]
H3K4me3	AML1, AML1/ETO and HEB	ChIP-qPCR	Cell lines	Gardini et al. [73]
K43me	AML1/ETO, EZH1	ChIP-Seq	Cell lines	Dou et al. [38]

The chromatin marks in Wang and Dou et al. refer to site-specific lysine acetylation/methylation of the respective target protein.

In summary, accumulating evidence implicates *AML1/ETO* as a potentially important epigenetic modifier similar to PML-RARα [54], activating and repressing gene transcription depending on the context of the interacting chromatin-modifying enzymes. These data support a novel mechanistic rationale encouraging the use of epigenetically active drugs such as HDAC and DNMT inhibitors, which are already in clinical use, to treat patients harboring the t(8;21) fusion. Future investigations could utilize compounds that specifically target transcriptional co-activators recruited by AML1/ETO, such as PRMT1 and P300, as a means of exploiting a vulnerability intrinsic to this leukemia subtype.

### Global changes in chromatin modifications, transcription factor binding, and gene expression mediated by AML1/ETO

A number of recent studies have performed global analyses on the ability of *AML1/ETO* to reorganize the chromatin and transcription factor binding landscape of human hematopoietic cells (see Table 1). Both AML1 and AML1/ETO localize in a multi-protein complex interacting with other transcription factors that together regulate differentiation of hematopoietic cells and leukemic blasts. The overexpression and depletion of single transcription factors redistributes the localization of this multi-protein complex creating novel binding sites [55]. AML1/ETO interacts with, and mutually stabilizes, CBFβ, E proteins like HEB and E2A, E-box-binding transcription factor LYL1, as well as LMO2 and its interacting partner LDB1 in a so-called AML1/ETO-containing transcription factor complex [55]. AML1/ETO competes for the same binding sites as AML1 and C/EBPα. Importantly, AML1/ETO negatively regulates the expression of C/EBPα by inhibiting positive autoregulation of the C/EBPα promoter [56, 57]. As a result, the selective depletion of AML1/ETO results in upregulation of *C/EBPα* and together with AML1 restores the differentiation-associated transcriptional program of leukemic cells through regulatory elements previously occupied by AML1/ETO [58]. Classically upregulated genes identified in t(8;21) leukemia include *p21/CDKNA1* [59, 60], *SOX4, IL-17BR, CD200*, and *γ-catenin* [61], and cytokine receptors like *CSF3R* [62]. Downregulated genes include cytokines such as *IL-6* [46] and *CSF2* [63], transcription factors such as *C/EBP*α [57] and proteins involved in cell cycle regulation like *CDKN2A* [64] (Table 2).

**Table 2:** Selected, clinically validated target genes of AML1/ETO (identified with the use of unbiased screening approaches).

Target gene	Function	Biological process	Reference
Upregulated			
SOX4, IL-17RB, CD200, and JUP (γ-catenin)	Transcription factor, cytokine receptor, anti-inflammatory signal, Adherens junction	Transcriptional regulation, inflammation, cell adhesion	Tonks et al. [61]
p21^waf1^ (CDKN1A)	Cyclin-dependent kinase inhibitor	Cell cycle, stem cell maintenance	Berg et al. [59], Peterson et al. [60]
BCL2^a^	Anti-apoptotic signal	Apoptosis	Martens et al. [66], Klampfer et al. [119]
FOXO1	Transcription factor	Apoptosis, stem cell maintenance	Lin et al. [120]
GFI1	Transcriptional repressor	Transcriptional regulation, G_1_/S-transition, oncogene	Marneth et al. [121]
TRKA (NTRK1)	MAPK pathway activation, protein kinase	Neuronal development, myeloid differentiation	Mulloy et al. [122]
ZFP36L1 (ERF-1, TIS11b)	Polypeptide chain release factor	mRNA translation	Shimada et al. [123]
ARG2, MT2A	Arginine metabolism, metal homeostasis	Immune response, oxidative Stress	Shia et al. [39]
CD48	NK-cell mediated immunosurveillance	Adaptive immune response, leukocyte migration	Wang et al. [37]
CSF3R	Cytokine receptor	Regulation of hematopoiesis	Shimizu et al. [62]
PAX5	Transcription factor	B-cell maturation	Tiacci et al. [124]
POU4F1	Transcription factor	Transcriptional regulation, B-lymphoid expression	Fortier et al. [125], Dunne et al. [126]
VLA4 (ITGA4)	Cell adhesion and migration	Leukocyte trafficking, Regulation of hematopoiesis	Ponnusamy et al. [127]
Downregulated			
IL-3	Cytokine	Regulation of hematopoiesis	Buchi et al. [46]
CSF2	Cytokine	Regulation of hematopoiesis	Frank et al. [63]
CCL3	Chemokine ligand	Chemotaxis, immune response	Bristow and Shore [128]
CEBPA	Transcription factor	Regulation of hematopoiesis	Koschmieder et al. [56] Pabst et al. [57]
LAT2	Adaptor molecule	Regulation of hematopoiesis	Fliegauf et al. [47] Duque-Afonso et al. [49, 52]; Essig [50]
p14^ARF^ (CDKN2A)^b^	Cyclin-dependent kinase inhibitor	Cell cycle, G_1_/S-transition, stem cell maintenance	Linggi et al. [64]
RASSF2	K-RAS-specific effector protein	Rac GTPase activation, Rac-mediated signal transduction	Stoner et al. [129]
Lysozyme (LYZ)	Bacteriolytic enzyme	Antimicrobial humoral response, myeloid differentiation	Claus et al. [130]
OGG1	DNA repair enzyme	Response to oxidative stress	Liddiard et al. [131]
PSGL1 (SELPLG)	Cell adhesion and migration	Leukocyte trafficking, regulation of hematopoiesis	Ponnusamy et al. [132]
NF1	GTPase-activating protein	Ras signal transduction	Yang et al. [133]
miR 144/451	Posttranscriptional regulation	Erythroid differentiation	Kohrs et al. [134]
SPI1 (PU.1)	Transcription factor	Regulation of hematopoiesis	Vangala et al. [135]

^a^Individual studies have demonstrated downregulation of BCL2 in leukemic cell lines [136].

^b^Referring to the alternate open reading frame (ARF) which does not function as a CDK4/6 inhibitor.

The chimeric fusion protein AML1/ETO not only modifies chromatin marks locally, as previously described on its target genes *IL-3* and *LAT2*, but also genome-wide. The effected gene expression and chromatin landscape is distinct from other oncogenic fusions harboring RUNX1 such as RUNX1-EVI1 [65]. E-twenty-six (ETS) family transcription factors such as *ERG* and *FLI1* guide and facilitate genome-wide binding of AML1/ETO as demonstrated in human cell lines and primary leukemic blasts. Binding of AML1/ETO to most *ERG* sites decreases acetylation of histone H3, H4, and of the specific residues H3K9 and H3K14, correlating with decreased gene expression [66]. AML1/ETO induces profound genome-wide changes and global gene transcriptional reprogramming by decreasing acetylation of H3K9 and RNA polymerase II (RNApol II) promoter occupancy. Interestingly, these epigenetic alterations are reversible at a global scale when *AML1/ETO* expression is altered, suggesting that targeting either function or expression of the fusion protein may represent a feasible therapeutic approach [58, 67]. Ptasinska et al. recently illustrated the importance of *AML1/ETO* expression levels, demonstrating that knockdown results in extensive changes in transcription factor binding and gene expression, and specifically to C/EBPα and AP-1 mediated alterations in promoter–enhancer interactions [68]. In sum, a multitude of groups have associated AML1/ETO with the recruitment of transcription factors and chromatin-modifying enzymes and consequently, genome-wide histone modifications, supporting the general role of AML1/ETO as an important epigenetic modifier in leukemia [69–73]. An overview of validated target genes of AML1/ETO that have been identified using unbiased screening approaches is provided in Table 2.

### The role of AML1/ETO in leukemogenesis

But how do the global changes in the epigenetic network facilitated by the AML1/ETO oncofusion protein contribute to leukemogenesis? Recently, Martinez-Soria et al. identified Cyclin D2 (*CCND2*) as a crucial transmitter of AML1/ETO-driven leukemic propagation, illustrating that AML1/ETO cooperates with AP-1 to drive *CCND2* expression, resulting in G_1_ cell cycle progression and leukemic propagation. The authors demonstrated that pharmacologic inhibition of CCND2 impaired leukemic expansion in a patient-derived AML model [74]. A further interdependency was identified for *TAF1*. Not only does knockdown of *TAF1* alter the association of AML1/ETO with chromatin, it is indeed required for leukemic cell self-renewal [75]. Moreover, reduction of *TAF1* promoted differentiation and apoptosis of AML cells harboring the AML1/ETO fusion, implicating the transcription factor as a potential therapeutic target. Leukemic growth was also demonstrated to be dependent on the DNA-binding protein MEIS2, as the co-expression of MEIS2 with AML1/ETO induced AML in a murine model [76]. An unconventional oncogenic partner in AML1/ETO-driven leukemic growth was identified in *HIF1*α, a transcription factor critical for the cellular response to oxygen depravation in malignant cells [77]. High *HIF1*α levels were correlated with increased AML1/ETO levels, and predicted inferior survival in t(8;21) AML patients.

Perturbations of the transcription factors *AML1* and *ERG* prevent the overexpression of the *AML1/ETO* oncogene and the onset of the apoptosis program in t(8;21) AMLs [70]. Importantly, targeted knockdown of *AML1* in AML1/ETO-positive cells decreases cell-cycling and induces apoptosis, suggesting that a fragile balance between AML1 and AML1/ETO must be maintained to sustain the malignant phenotype [78]. Together these promising findings highlight potential therapeutic vulnerabilities exposed by the dependency of AML1/ETO-driven leukemogenesis on other transcription factors and mediators of cell cycle progression. Novel experimental techniques such as differentiation models utilizing induced pluripotent stem cells, are now being employed to further model AML1/ETO induced oncogenesis [79].

### Cooperating oncogenic events in AML1/ETO positive AML

**Fig. 3:**
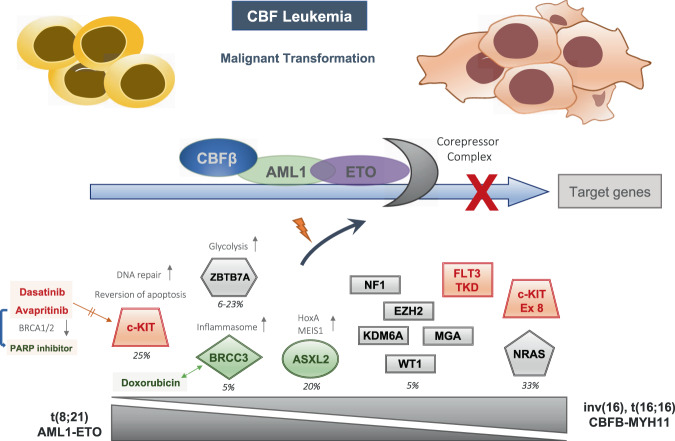
Cooperating genetic lesions contribute to AML1/ETO-driven leukemogenesis. Gene mutations conferring a positive prognostic impact are highlighted in green, while gene mutations with a poor prognostic are indicated in red. Gene mutations with an equivocal prognostic impact are shown in gray. The relative incidence of the respective mutation is depicted in percent. Mutations occurring more frequently in t(8;21) CBF-AML are portrayed to the left, while mutations with a preponderance in inv(16)/t(16;16) CBF-AML are portrayed to the right.

In the last decades, CBF leukemias have served as a crucial model for the two-hit hypothesis of leukemogenesis. Using unbiased approaches based on next-generation sequencing technologies, several mutations have now been identified in CBF leukemias that play a cooperative role in promoting leukemia (Fig. 3) [8, 80]. Overall, at least one additional mutation was identified in 95% of t(8;21) patients, with a mean of 2.2 driver mutations per patient [81].

Approximately two-thirds of CBF leukemia cases harbor activating mutations in *NRAS, KIT, FLT3, KRAS, PTPN11*, and/or loss-of-function mutations in *NF1* [80]. Importantly, the mutational load at diagnosis is prognostic, as patients with a higher burden of co-mutations have a significantly higher relapse rate with a trend towards inferior survival [82]. While *NRAS* represents the most frequently mutated gene in CBF leukemias—mutated in one-third of all patients—it is not associated with a worse clinical outcome. A relative preponderance of the *NRAS* mutation was observed in inv(16) compared to t(8;21) leukemia. Moreover, the spectrum of *NRAS* mutations differed between both CBF leukemias, and exon 61 mutations were more frequently observed in *CBFB/MYH11* AMLs (i.e., harboring inv(16) or t(16;16)).

*KIT* mutations are present in about 25% of CBF leukemias and are associated with inferior outcomes [81, 83]. The *KIT* exon 17 mutation is particularly enriched in *AML1/*ETO-positive leukemias. In a large study of *CBFB/MYH11* AMLs, both the c-*KIT* exon 8 and *FLT3-TKD* mutations represented markers of poor prognosis [84]. Interestingly, AML1/ETO epigenetically trans-activates c-*KIT* expression by binding and then recruiting the histone acetyltransferase P300 to the c-*KIT* promoter [85, 86]. Activating c-*KIT* mutations confer oncogenic cooperativity by augmenting DNA repair and reverting apoptosis, offering a potential mechanistic explanation for the increased chemo-resistance observed in t(8;21) patients with cooperating c-*KIT* mutations [87]. Using *KIT* as a therapeutic target, addition of the multi-receptor tyrosine kinase dasatinib to conventional chemotherapy demonstrated a safe profile and promising efficacy in a phase Ib/IIa clinical trial [88]. Early in vivo studies suggest a mechanism wherein dasatinib can induce differentiation of t(8;21) AML blasts into neutrophilic granulocytes [89]. A promising recent report revealed that inhibition of mutated c-*KIT* using avapritinib in AML1/ETO-positive leukemia restored sensitivity to PARP inhibition via downregulation of *BRCA1/*2 [90].

Among epigenetic regulators, the polycomb-associated protein *ASXL2* is mutated in about 20% of AML1/ETO-positive pediatric and adult patients [80, 91]. While recurrent gene mutations have a positive prognostic impact in children [92], the results were equivocal in adults [93]. In a murine model studying *ASXL2* loss in the context of the AML1/ETO oncofusion, *ASXL2* functions as a haploinsufficient tumor suppressor when mice are challenged with either the full-length (AE) or short (AE9a) splice isoform of *AML1/ETO* [94]. Mechanistically, ASXL2, AML1 and AML1/ETO displayed an overlap in target gene expression. While global chromatin accessibility was not altered between conditions, a significant increase in chromatin accessibility at putative enhancers of key leukemogenic loci including *HoxA* and *Meis1* was observed in mice lacking *Asxl2* [94].

The transcription factor *ZBTB7A* is recurrently mutated in 6–23% of t(8;21) AML patients and is rarely found in *CBFB/MYH11* AMLs [80, 95, 96]. To date, a prognostic impact of the mutation on clinical outcomes has not been demonstrated. The mutation disrupts the transcriptional repressor potential and anti-proliferative effect of *ZBTB7A* [96]. A potential mechanism includes the de-repression of glycolytic genes upon *ZBTB7A* deletion or mutation, which results in increased glycolysis, and thus provides more energy to the tumor cell [97, 98]. However, this addiction to glycolysis may be exploited therapeutically, as a recent study demonstrated that loss of *ZBTB7A* sensitized leukemic blasts to metabolic inhibition with 2-deoxy-D-glucose [99].

The mutation in the lysine 63-specific deubiquitinating enzyme *BRCC3*—found selectively in about 5% of t(8;21) AMLs and associated with excellent clinical outcomes—was recently functionally characterized [100]. *BRCC3* mutations resulted in an impaired interferon response and diminished inflammasome activity. This may abrogate the strong activation of interferon signaling conferred by AML1/ETO, which has been demonstrated to negatively affect the leukemic potential of the oncofusion [101]. On a cellular level, the inactivation of BRCC3 led to a higher sensitivity to doxorubicin due to an impaired DNA damage response, offering an explanation for the favorable outcomes of *BRCC3* mutated AML patients. Other genes recurrently mutated in about 5% of CBF leukemias include the transcription factors *WT1* and *MGA*, and the epigenetic regulators *EZH2* and *KDM6A* [80]. Further mechanistic studies are required to delineate the functional implications of cooperating and competing mutations in the pathogenesis of *AML1/ETO* leukemia.

### Conclusions and outlook

Despite more than two decades of preclinical and translational research on AML1/ETO, there still remain a number of open questions that need to be addressed in order to introduce novel therapeutic approaches into the treatment of patients with t(8;21) positive AML. We have learned that gene repression by AML1/ETO is mediated via HDAC activity. While early preclinical studies demonstrated antineoplastic activity for HDAC inhibitors [102, 103] and hypomethylating agents [104] in AML1/ETO-expressing cells, the clinical use of HDAC inhibitors in AML has been disappointing to date [105–107]. These poor response rates do not support further development of this approach. Possibly, other, as yet unidentified chromatin-modifying enzymes are also involved in the pathogenesis of the disease, and drugs targeting these may yield superior results.

The “high-hanging fruit” remains direct disruption of the AML1/ETO recruited protein complex, though DNA-binding proteins have historically been difficult to target due to a lack of high-throughput screening methods [108]. While challenging, potential therapeutic strategies include exploiting the stability of the mutant oncoprotein either by targeting molecular chaperones (e.g., *Calpain B* or *Hsp90*), serine proteases (e.g., *Cathepsin G*), or via proteasome inhibitors like bortezomib [109–113]. The development of Runt domain inhibitors (RDIs), which disrupt CBF binding and function, represents a further promising approach [114]. Targeting the posttranslational modifications that control the function of the oncofusion represents an alternative concept. For example, the site-specific acetylation of the NHR1 domain of ETO facilitated by *P300* could be inhibited by means of RNA interference or chemical inhibition. Both in vivo and in vitro models demonstrated reduced levels of effector proteins required for cell renewal upon *P300* inhibition, pointing towards *P300* as an attractive drug target [36]. Recently, Yang et al. report the development of an oral *P300/CBP* histone acetyltransferase inhibitor using an artificial-intelligence-assisted drug discovery pipeline, which demonstrated efficacy in preclinical studies [115].

However, the broad landscape of cooperating genetic lesions makes it unlikely that directly targeting the *AML1/ETO* recruited multi-protein complex represents the panacea of CBF leukemia. Novel therapeutic strategies must navigate this individual genetic landscape, taking advantage of the interactive proteins, epigenetic mechanisms, and molecular pathways that jointly drive the neoplastic transformation of hematopoietic cells. The addition of the multikinase inhibitor dasatinib to a conventional chemotherapy backbone represents a prominent example of such an approach—exploiting the frequent occurrence of *KIT* mutations and higher *KIT* expression levels in CBF leukemia. Early clinical trial data has been encouraging, demonstrating excellent outcomes for both younger and older patients irrespective of *KIT* mutational status [88, 116]. These data have prompted a large randomized Phase III trial that is currently ongoing (NCT02013658). Depending on the outcome of c-KIT inhibition, further development of this indirect targeting of hematopoietic function using more specific and potent KIT inhibitors may be warranted. The fact that AML1/ETO-driven disease depends on cyclin D2 may confer a therapeutic avenue for palbociclib, a selective inhibitor of the cyclin-dependent kinases CDK4 and CDK6 [74, 117]. As synergistic effects were observed upon addition of a tyrosine kinase inhibitor, combinatorial strategies may enhance therapeutic efficacy.

The discovery and validation of multiple, biologically highly relevant target genes of AML1/ETO underlines the importance of this leukemia as a very useful model to study the function of a chimeric transcription factor oncogene. Elucidating the mechanism of epigenetic regulation at the heart of this disease entity will be critical to achieve the long-term goal of further improving the prognosis of patients afflicted with this cancer.

## References

[1] Döhner H, Weisdorf DJ, Bloomfield CD. Acute myeloid leukemia. N Engl. J. Med. 2015;373:1136–52. 10.1056/NEJMra1406184 26376137

[2] Tyner JW, Tognon CE, Bottomly D, Wilmot B, Kurtz SE, Savage SL (2018). Functional genomic landscape of acute myeloid leukaemia. Nature..

[3] Papaemmanuil E, Gerstung M, Bullinger L, Gaidzik VI, Paschka P, Roberts ND (2016). Genomic classification and prognosis in acute myeloid leukemia. N Engl J Med..

[4] DiNardo CD, Stein EM, de Botton S, Roboz GJ, Altman JK, Mims AS (2018). Durable remissions with ivosidenib in IDH1-mutated relapsed or refractory AML. N Engl J Med..

[5] Stein EM, DiNardo CD, Pollyea DA, Fathi AT, Roboz GJ, Altman JK (2017). Enasidenib in mutant IDH2 relapsed or refractory acute myeloid leukemia. Blood..

[6] Rowley JD (1973). Identificaton of a translocation with quinacrine fluorescence in a patient with acute leukemia. Ann Genet..

[7] Bolouri H, Farrar JE, Triche T, Ries RE, Lim EL, Alonzo TA (2018). The molecular landscape of pediatric acute myeloid leukemia reveals recurrent structural alterations and age-specific mutational interactions. Nat Med..

[8] Haferlach T, Meggendorfer M (2019). More than a fusion gene: the RUNX1-RUNX1T1 AML. Blood..

[9] Yin JA, O’Brien MA, Hills RK, Daly SB, Wheatley K, Burnett AK (2012). Minimal residual disease monitoring by quantitative RT-PCR in core binding factor AML allows risk stratification and predicts relapse: results of the United Kingdom MRC AML-15 trial. Blood..

[10] Lorsbach RB, Moore J, Ang SO, Sun W, Lenny N, Downing JR (2004). Role of RUNX1 in adult hematopoiesis: analysis of RUNX1-IRES-GFP knock-in mice reveals differential lineage expression. Blood..

[11] Sood R, Kamikubo Y, Liu P (2017). Role of RUNX1 in hematological malignancies. Blood..

[12] Cai Z, de Bruijn M, Ma X, Dortland B, Luteijn T, Downing RJ (2000). Haploinsufficiency of AML1 affects the temporal and spatial generation of hematopoietic stem cells in the mouse embryo. Immunity..

[13] Bruhn L, Munnerlyn A, Grosschedl R (1997). ALY, a context-dependent coactivator of LEF-1 and AML-1, is required for TCRalpha enhancer function. Genes Dev..

[14] Licht JD (2001). AML1 and the AML1-ETO fusion protein in the pathogenesis of t(8;21) AML. Oncogene..

[15] Zhang J, Kalkum M, Yamamura S, Chait BT, Roeder RG (2004). E protein silencing by the leukemogenic AML1-ETO fusion protein. Science..

[16] Liu Y, Cheney MD, Gaudet JJ, Chruszcz M, Lukasik SM, Sugiyama D (2006). The tetramer structure of the Nervy homology two domain, NHR2, is critical for AML1/ETO’s activity. Cancer Cell..

[17] Zhang J, Hug BA, Huang EY, Chen CW, Gelmetti V, Maccarana M (2001). Oligomerization of ETO is obligatory for corepressor interaction. Mol Cell Biol.

[18] Lenny N, Meyers S, Hiebert SW (1995). Functional domains of the t(8;21) fusion protein, AML-1/ETO. Oncogene..

[19] Lutterbach B, Sun D, Schuetz J, Hiebert SW (1998). The MYND motif is required for repression of basal transcription from the multidrug resistance 1 promoter by the t(8;21) fusion protein. Mol Cell Biol..

[20] Gelmetti V, Zhang J, Fanelli M, Minucci S, Pelicci PG, Lazar MA (1998). Aberrant recruitment of the nuclear receptor corepressor-histone deacetylase complex by the acute myeloid leukemia fusion partner ETO. Mol Cell Biol..

[21] Solari L, Bauer T, Dicker F, Haferlach C, Griesshammer M, Schnittger S (2013). A novel recurrent AML1-ETO fusion: tight in vivo association with BCR-ABL1. Leukemia..

[22] De Braekeleer E, Douet-Guilbert N, Morel F, Le Bris MJ, Ferec C, De Braekeleer M (2011). RUNX1 translocations and fusion genes in malignant hemopathies. Future Oncol.

[23] Lausten-Thomsen U, Madsen HO, Vestergaard TR, Hjalgrim H, Nersting J, Schmiegelow K (2011). Prevalence of t(12;21)[ETV6-RUNX1]-positive cells in healthy neonates. Blood..

[24] Higuchi M, O’Brien D, Kumaravelu P, Lenny N, Yeoh EJ, Downing JR (2002). Expression of a conditional AML1-ETO oncogene bypasses embryonic lethality and establishes a murine model of human t(8;21) acute myeloid leukemia. Cancer Cell.

[25] Yan M, Burel SA, Peterson LF, Kanbe E, Iwasaki H, Boyapati A (2004). Deletion of an AML1-ETO C-terminal NcoR/SMRT-interacting region strongly induces leukemia development. Proc Natl Acad Sci USA..

[26] Wolford JK, Prochazka M (1998). Structure and expression of the human MTG8/ETO gene. Gene..

[27] Yan M, Kanbe E, Peterson LF, Boyapati A, Miao Y, Wang Y (2006). A previously unidentified alternatively spliced isoform of t(8;21) transcript promotes leukemogenesis. Nat Med..

[28] Kozu T, Fukuyama T, Yamami T, Akagi K, Kaneko Y (2005). MYND-less splice variants of AML1-MTG8 (RUNX1-CBFA2T1) are expressed in leukemia with t(8;21). Genes Chromosomes Cancer..

[29] Mannari D, Gascoyne D, Dunne J, Chaplin T, Young B (2010). A novel exon in AML1-ETO negatively influences the clonogenic potential of the t(8;21) in acute myeloid leukemia. Leukemia..

[30] LaFiura KM, Edwards H, Taub JW, Matherly LH, Fontana JA, Mohamed AN (2008). Identification and characterization of novel AML1-ETO fusion transcripts in pediatric t(8;21) acute myeloid leukemia: a report from the Children’s Oncology Group. Oncogene..

[31] Duncavage EJ, Schroeder MC, O’Laughlin M, Wilson R, MacMillan S, Bohannon A (2021). Genome sequencing as an alternative to cytogenetic analysis in myeloid cancers. N Engl J Med..

[32] Qiu J, Zhou B, Thol F, Zhou Y, Chen L, Shao C (2016). Distinct splicing signatures affect converged pathways in myelodysplastic syndrome patients carrying mutations in different splicing regulators. RNA..

[33] Komeno Y, Yan M, Matsuura S, Lam K, Lo MC, Huang YJ (2014). Runx1 exon 6-related alternative splicing isoforms differentially regulate hematopoiesis in mice. Blood..

[34] Grinev VV, Barneh F, Ilyushonak IM, Nakjang S, Smink J, van Oort A (2021). RUNX1/RUNX1T1 mediates alternative splicing and reorganises the transcriptional landscape in leukemia. Nat Commun..

[35] Choudhary C, Kumar C, Gnad F, Nielsen ML, Rehman M, Walther TC (2009). Lysine acetylation targets protein complexes and co-regulates major cellular functions. Science..

[36] Wang L, Gural A, Sun XJ, Zhao X, Perna F, Huang G (2011). The leukemogenicity of AML1-ETO is dependent on site-specific lysine acetylation. Science..

[37] Wang Z, Guan W, Wang M, Chen J, Zhang L, Xiao Y, et al. AML1-ETO inhibits acute myeloid leukemia immune escape by CD48. Leuk Lymphoma. 2021;62:937–43. 10.1080/10428194.2020.1849680 33225787

[38] Dou L, Yan F, Pang J, Zheng D, Li D, Gao L (2019). Protein lysine 43 methylation by EZH1 promotes AML1-ETO transcriptional repression in leukemia. Nat Commun.

[39] Shia WJ, Okumura AJ, Yan M, Sarkeshik A, Lo MC, Matsuura S (2012). PRMT1 interacts with AML1-ETO to promote its transcriptional activation and progenitor cell proliferative potential. Blood..

[40] Zhao B, Ye X, Yu J, Li L, Li W, Li S (2008). TEAD mediates YAP-dependent gene induction and growth control. Genes Dev..

[41] Amann JM, Nip J, Strom DK, Lutterbach B, Harada H, Lenny N (2001). ETO, a target of t(8;21) in acute leukemia, makes distinct contacts with multiple histone deacetylases and binds mSin3A through its oligomerization domain. Mol Cell Biol..

[42] Hildebrand D, Tiefenbach J, Heinzel T, Grez M, Maurer AB (2001). Multiple regions of ETO cooperate in transcriptional repression. J Biol Chem.

[43] Liu S, Shen T, Huynh L, Klisovic MI, Rush LJ, Ford JL (2005). Interplay of RUNX1/MTG8 and DNA methyltransferase 1 in acute myeloid leukemia. Cancer Res..

[44] Liu S, Klisovic RB, Vukosavljevic T, Yu J, Paschka P, Huynh L (2007). Targeting AML1/ETO-histone deacetylase repressor complex: a novel mechanism for valproic acid-mediated gene expression and cellular differentiation in AML1/ETO-positive acute myeloid leukemia cells. J Pharmacol Exp Ther..

[45] Fazi F, Zardo G, Gelmetti V, Travaglini L, Ciolfi A, Di Croce L (2007). Heterochromatic gene repression of the retinoic acid pathway in acute myeloid leukemia. Blood..

[46] Buchi F, Masala E, Rossi A, Valencia A, Spinelli E, Sanna A (2014). Redistribution of H3K27me3 and acetylated histone H4 upon exposure to azacitidine and decitabine results in de-repression of the AML1/ETO target gene IL3. Epigenetics..

[47] Fliegauf M, Stock M, Berg T, Lübbert M (2004). Williams-Beuren syndrome critical region-5/non-T-cell activation linker: a novel target gene of AML1/ETO. Oncogene..

[48] Teppo S, Laukkanen S, Liuksiala T, Nordlund J, Oittinen M, Teittinen K (2016). Genome-wide repression of eRNA and target gene loci by the ETV6-RUNX1 fusion in acute leukemia. Genome Res..

[49] Duque-Afonso J, Solari L, Essig A, Berg T, Pahl HL, Lübbert M (2011). Regulation of the adaptor molecule LAT2, an in vivo target gene of AML1/ETO (RUNX1/RUNX1T1), during myeloid differentiation. Br J Haematol..

[50] Essig A, Duque-Afonso J, Schwemmers S, Pahl HL, Lübbert M (2014). The AML1/ETO target gene LAT2 interferes with differentiation of normal hematopoietic precursor cells. Leuk Res..

[51] Thome CH, Ferreira GA, Pereira-Martins DA, Dos Santos GA, Ortiz CA, de Souza LEB (2020). NTAL is associated with treatment outcome, cell proliferation and differentiation in acute promyelocytic leukemia. Sci Rep..

[52] Duque-Afonso J, Yalcin A, Berg T, Abdelkarim M, Heidenreich O, Lübbert M (2011). The HDAC class I-specific inhibitor entinostat (MS-275) effectively relieves epigenetic silencing of the LAT2 gene mediated by AML1/ETO. Oncogene..

[53] Viré E, Brenner C, Deplus R, Blanchon L, Fraga M, Didelot C (2006). The Polycomb group protein EZH2 directly controls DNA methylation. Nature..

[54] Di Croce L, Raker VA, Corsaro M, Fazi F, Fanelli M, Faretta M (2002). Methyltransferase recruitment and DNA hypermethylation of target promoters by an oncogenic transcription factor. Science..

[55] Sun XJ, Wang Z, Wang L, Jiang Y, Kost N, Soong TD (2013). A stable transcription factor complex nucleated by oligomeric AML1-ETO controls leukaemogenesis. Nature..

[56] Koschmieder S, Halmos B, Levantini E, Tenen DG (2009). Dysregulation of the C/EBPalpha differentiation pathway in human cancer. J Clin Oncol.

[57] Pabst T, Mueller BU, Harakawa N, Schoch C, Haferlach T, Behre G (2001). AML1-ETO downregulates the granulocytic differentiation factor C/EBPalpha in t(8;21) myeloid leukemia. Nat Med..

[58] Ptasinska A, Assi SA, Martinez-Soria N, Imperato MR, Piper J, Cauchy P (2014). Identification of a dynamic core transcriptional network in t(8;21) AML that regulates differentiation block and self-renewal. Cell Rep..

[59] Berg T, Fliegauf M, Burger J, Staege MS, Liu S, Martinez N (2008). Transcriptional upregulation of p21/WAF/Cip1 in myeloid leukemic blasts expressing AML1-ETO. Haematologica..

[60] Peterson LF, Yan M, Zhang DE (2007). The p21Waf1 pathway is involved in blocking leukemogenesis by the t(8;21) fusion protein AML1-ETO. Blood..

[61] Tonks A, Pearn L, Musson M, Gilkes A, Mills KI, Burnett AK (2007). Transcriptional dysregulation mediated by RUNX1-RUNX1T1 in normal human progenitor cells and in acute myeloid leukaemia. Leukemia..

[62] Shimizu K, Kitabayashi I, Kamada N, Abe T, Maseki N, Suzukawa K (2000). AML1-MTG8 leukemic protein induces the expression of granulocyte colony-stimulating factor (G-CSF) receptor through the up-regulation of CCAAT/enhancer binding protein epsilon. Blood..

[63] Frank R, Zhang J, Uchida H, Meyers S, Hiebert SW, Nimer SD (1995). The AML1/ETO fusion protein blocks transactivation of the GM-CSF promoter by AML1B. Oncogene..

[64] Linggi B, Müller-Tidow C, van de Locht L, Hu M, Nip J, Serve H (2002). The t(8;21) fusion protein, AML1 ETO, specifically represses the transcription of the p14(ARF) tumor suppressor in acute myeloid leukemia. Nat Med..

[65] Loke J, Assi SA, Imperato MR, Ptasinska A, Cauchy P, Grabovska Y (2017). RUNX1-ETO and RUNX1-EVI1 differentially reprogram the chromatin landscape in t(8;21) and t(3;21) AML. Cell Rep..

[66] Martens JH, Mandoli A, Simmer F, Wierenga BJ, Saeed S, Singh AA (2012). ERG and FLI1 binding sites demarcate targets for aberrant epigenetic regulation by AML1-ETO in acute myeloid leukemia. Blood..

[67] Ptasinska A, Assi SA, Mannari D, James SR, Williamson D, Dunne J (2012). Depletion of RUNX1/ETO in t(8;21) AML cells leads to genome-wide changes in chromatin structure and transcription factor binding. Leukemia..

[68] Ptasinska A, Pickin A, Assi SA, Chin PS, Ames L, Avellino R (2019). RUNX1-ETO depletion in t(8;21) AML leads to C/EBPalpha- and AP-1-mediated alterations in enhancer-promoter interaction. Cell Rep..

[69] Saeed S, Logie C, Francoijs KJ, Frige G, Romanenghi M, Nielsen FG (2012). Chromatin accessibility, p300, and histone acetylation define PML-RARalpha and AML1-ETO binding sites in acute myeloid leukemia. Blood..

[70] Mandoli A, Singh AA, Prange KHM, Tijchon E, Oerlemans M, Dirks R (2016). The hematopoietic transcription factors RUNX1 and ERG prevent AML1-ETO oncogene overexpression and onset of the apoptosis program in t(8;21) AMLs. Cell Rep..

[71] Li Y, Wang H, Wang X, Jin W, Tan Y, Fang H (2016). Genome-wide studies identify a novel interplay between AML1 and AML1/ETO in t(8;21) acute myeloid leukemia. Blood..

[72] Trombly DJ, Whitfield TW, Padmanabhan S, Gordon JA, Lian JB, van Wijnen AJ (2015). Genome-wide co-occupancy of AML1-ETO and N-CoR defines the t(8;21) AML signature in leukemic cells. BMC Genom..

[73] Gardini A, Cesaroni M, Luzi L, Okumura AJ, Biggs JR, Minardi SP (2008). AML1/ETO oncoprotein is directed to AML1 binding regions and co-localizes with AML1 and HEB on its targets. PLoS Genet..

[74] Martinez-Soria N, McKenzie L, Draper J, Ptasinska A, Issa H, Potluri S (2018). The oncogenic transcription factor RUNX1/ETO corrupts cell cycle regulation to drive leukemic transformation. Cancer Cell..

[75] Xu Y, Man N, Karl D, Martinez C, Liu F, Sun J (2019). TAF1 plays a critical role in AML1-ETO driven leukemogenesis. Nat Commun..

[76] Vegi NM, Klappacher J, Oswald F, Mulaw MA, Mandoli A, Thiel VN (2016). MEIS2 is an oncogenic partner in AML1-ETO-positive AML. Cell Rep..

[77] Gao XN, Yan F, Lin J, Gao L, Lu XL, Wei SC (2015). AML1/ETO cooperates with HIF1alpha to promote leukemogenesis through DNMT3a transactivation. Leukemia..

[78] Ben-Ami O, Friedman D, Leshkowitz D, Goldenberg D, Orlovsky K, Pencovich N (2013). Addiction of t(8;21) and inv(16) acute myeloid leukemia to native RUNX1. Cell Rep..

[79] Tijchon E, Yi G, Mandoli A, Smits JGA, Ferrari F, Heuts BMH (2019). The acute myeloid leukemia associated AML1-ETO fusion protein alters the transcriptome and cellular progression in a single-oncogene expressing in vitro induced pluripotent stem cell based granulocyte differentiation model. PLoS ONE..

[80] Faber ZJ, Chen X, Gedman AL, Boggs K, Cheng J, Ma J (2016). The genomic landscape of core-binding factor acute myeloid leukemias. Nat Genet..

[81] Christen F, Hoyer K, Yoshida K, Hou HA, Waldhueter N, Heuser M (2019). Genomic landscape and clonal evolution of acute myeloid leukemia with t(8;21): an international study on 331 patients. Blood..

[82] Hollein A, Nadarajah N, Meggendorfer M, Jeromin S, Kern W, Haferlach C (2019). Molecular characterization of AML with RUNX1-RUNX1T1 at diagnosis and relapse reveals net loss of co-mutations. Hemasphere..

[83] Paschka P, Marcucci G, Ruppert AS, Mrozek K, Chen H, Kittles RA (2006). Adverse prognostic significance of KIT mutations in adult acute myeloid leukemia with inv(16) and t(8;21): a Cancer and Leukemia Group B Study. J Clin Oncol..

[84] Paschka P, Du J, Schlenk RF, Gaidzik VI, Bullinger L, Corbacioglu A (2013). Secondary genetic lesions in acute myeloid leukemia with inv(16) or t(16;16): a study of the German-Austrian AML Study Group (AMLSG). Blood..

[85] Chen G, Liu A, Xu Y, Gao L, Jiang M, Li Y (2019). The RUNX1-ETO fusion protein trans-activates c-KIT expression by recruiting histone acetyltransferase P300 on its promoter. FEBS J..

[86] Tian Y, Wang G, Hu Q, Xiao X, Chen S (2018). AML1/ETO trans-activates c-KIT expression through the long range interaction between promoter and intronic enhancer. J Cell Biochem..

[87] Wichmann C, Quagliano-Lo Coco I, Yildiz O, Chen-Wichmann L, Weber H, Syzonenko T (2015). Activating c-KIT mutations confer oncogenic cooperativity and rescue RUNX1/ETO-induced DNA damage and apoptosis in human primary CD34+ hematopoietic progenitors. Leukemia..

[88] Paschka P, Schlenk RF, Weber D, Benner A, Bullinger L, Heuser M (2018). Adding dasatinib to intensive treatment in core-binding factor acute myeloid leukemia-results of the AMLSG 11-08 trial. Leukemia..

[89] Chevalier N, Solari ML, Becker H, Pantic M, Gartner F, Maul-Pavicic A (2010). Robust in vivo differentiation of t(8;21)-positive acute myeloid leukemia blasts to neutrophilic granulocytes induced by treatment with dasatinib. Leukemia..

[90] Nieborowska-Skorska M, Paietta EM, Levine RL, Fernandez HF, Tallman MS, Litzow MR (2019). Inhibition of the mutated c-KIT kinase in AML1-ETO-positive leukemia cells restores sensitivity to PARP inhibitor. Blood Adv..

[91] Micol JB, Duployez N, Boissel N, Petit A, Geffroy S, Nibourel O (2014). Frequent ASXL2 mutations in acute myeloid leukemia patients with t(8;21)/RUNX1-RUNX1T1 chromosomal translocations. Blood..

[92] Yamato G, Shiba N, Yoshida K, Shiraishi Y, Hara Y, Ohki K (2017). ASXL2 mutations are frequently found in pediatric AML patients with t(8;21)/ RUNX1-RUNX1T1 and associated with a better prognosis. Genes Chromosomes Cancer..

[93] Jahn N, Agrawal M, Bullinger L, Weber D, Corbacioglu A, Gaidzik VI (2017). Incidence and prognostic impact of ASXL2 mutations in adult acute myeloid leukemia patients with t(8;21)(q22;q22): a study of the German-Austrian AML Study Group. Leukemia..

[94] Micol JB, Pastore A, Inoue D, Duployez N, Kim E, Lee SC (2017). ASXL2 is essential for haematopoiesis and acts as a haploinsufficient tumour suppressor in leukemia. Nat Commun.

[95] Lavallee VP, Lemieux S, Boucher G, Gendron P, Boivin I, Armstrong RN (2016). RNA-sequencing analysis of core binding factor AML identifies recurrent ZBTB7A mutations and defines RUNX1-CBFA2T3 fusion signature. Blood..

[96] Hartmann L, Dutta S, Opatz S, Vosberg S, Reiter K, Leubolt G (2016). ZBTB7A mutations in acute myeloid leukaemia with t(8;21) translocation. Nat Commun..

[97] Liu XS, Haines JE, Mehanna EK, Genet MD, Ben-Sahra I, Asara JM (2014). ZBTB7A acts as a tumor suppressor through the transcriptional repression of glycolysis. Genes Dev..

[98] Liu XS, Liu Z, Gerarduzzi C, Choi DE, Ganapathy S, Pandolfi PP (2016). Somatic human ZBTB7A zinc finger mutations promote cancer progression. Oncogene..

[99] Redondo Monte E, Wilding A, Leubolt G, Kerbs P, Bagnoli JW, Hartmann L (2020). ZBTB7A prevents RUNX1-RUNX1T1-dependent clonal expansion of human hematopoietic stem and progenitor cells. Oncogene..

[100] Meyer T, Jahn N, Lindner S, Rohner L, Dolnik A, Weber D (2020). Functional characterization of BRCC3 mutations in acute myeloid leukemia with t(8;21)(q22;q22.1). Leukemia..

[101] DeKelver RC, Lewin B, Weng S, Yan M, Biggs J, Zhang DE (2014). RUNX1-ETO induces a type I interferon response which negatively effects t(8;21)-induced increased self-renewal and leukemia development. Leuk Lymphoma..

[102] Tabe Y, Jin L, Contractor R, Gold D, Ruvolo P, Radke S (2007). Novel role of HDAC inhibitors in AML1/ETO AML cells: activation of apoptosis and phagocytosis through induction of annexin A1. Cell Death Differ..

[103] Cameron EE, Bachman KE, Myohanen S, Herman JG, Baylin SB (1999). Synergy of demethylation and histone deacetylase inhibition in the re-expression of genes silenced in cancer. Nat Genet..

[104] Berg T, Guo Y, Abdelkarim M, Fliegauf M, Lübbert M (2007). Reversal of p15/INK4b hypermethylation in AML1/ETO-positive and -negative myeloid leukemia cell lines. Leuk Res.

[105] Prebet T, Sun Z, Figueroa ME, Ketterling R, Melnick A, Greenberg PL (2014). Prolonged administration of azacitidine with or without entinostat for myelodysplastic syndrome and acute myeloid leukemia with myelodysplasia-related changes: results of the US Leukemia Intergroup trial E1905. J Clin Oncol.

[106] Craddock CF, Houlton AE, Quek LS, Ferguson P, Gbandi E, Roberts C (2017). Outcome of azacitidine therapy in acute myeloid leukemia is not improved by concurrent vorinostat therapy but is predicted by a diagnostic molecular signature. Clin Cancer Res.

[107] Lübbert M, Grishina O, Schmoor C, Schlenk RF, Jost E, Crysandt M (2020). Valproate and retinoic acid in combination with decitabine in elderly nonfit patients with acute myeloid leukemia: results of a multicenter, randomized, 2 x 2, Phase II trial. J Clin Oncol..

[108] Shiroma Y, Takahashi RU, Yamamoto Y, Tahara H, Targeting DNA (2020). binding proteins for cancer therapy. Cancer Sci.

[109] Tian WL, He F, Fu X, Lin JT, Tang P, Huang YM (2014). High expression of heat shock protein 90 alpha and its significance in human acute leukemia cells. Gene..

[110] Qi X, Zhang X, Liu X, Tang W, Dai J, Chen A (2021). HDN-1 induces cell differentiation toward apoptosis in promyelocytic leukemia cells depending on its selective effect on client proteins of Hsp90. Toxicol Appl Pharmacol.

[111] Jin W, Wu K, Li YZ, Yang WT, Zou B, Zhang F (2013). AML1-ETO targets and suppresses cathepsin G, a serine protease, which is able to degrade AML1-ETO in t(8;21) acute myeloid leukemia. Oncogene..

[112] Fang HT, Zhang B, Pan XF, Gao L, Zhen T, Zhao HX (2012). Bortezomib interferes with C-KIT processing and transforms the t(8;21)-generated fusion proteins into tumor-suppressing fragments in leukemia cells. Proc Natl Acad Sci USA..

[113] Arora R, Sawney S, Saluja D (2016). Potential therapeutic approaches for the treatment of acute myeloid leukemia with AML1-ETO translocation. Curr Cancer Drug Targets.

[114] Oo ZM, Illendula A, Grembecka J, Schmidt C, Zhou Y, Esain V (2018). A tool compound targeting the core binding factor Runt domain to disrupt binding to CBFbeta in leukemic cells. Leuk Lymphoma..

[115] Yang Y, Zhang R, Li Z, Mei L, Wan S, Ding H (2020). Discovery of highly potent, selective, and orally efficacious p300/CBP histone acetyltransferases inhibitors. J Med Chem..

[116] Marcucci G, Geyer S, Laumann K, Zhao W, Bucci D, Uy GL (2020). Combination of dasatinib with chemotherapy in previously untreated core binding factor acute myeloid leukemia: CALGB 10801. Blood Adv..

[117] Finn RS, Dering J, Conklin D, Kalous O, Cohen DJ, Desai AJ (2009). PD 0332991, a selective cyclin D kinase 4/6 inhibitor, preferentially inhibits proliferation of luminal estrogen receptor-positive human breast cancer cell lines in vitro. Breast Cancer Res..

[118] Maiques-Diaz A, Chou FS, Wunderlich M, Gomez-Lopez G, Jacinto FV, Rodriguez-Perales S (2012). Chromatin modifications induced by the AML1-ETO fusion protein reversibly silence its genomic targets through AML1 and Sp1 binding motifs. Leukemia..

[119] Klampfer L, Zhang J, Zelenetz AO, Uchida H, Nimer SD (1996). The AML1/ETO fusion protein activates transcription of BCL-2. Proc Natl Acad Sci USA..

[120] Lin S, Ptasinska A, Chen X, Shrestha M, Assi SA, Chin PS (2017). A FOXO1-induced oncogenic network defines the AML1-ETO preleukemic program. Blood..

[121] Marneth AE, Botezatu L, Hones JM, Israel JCL, Schutte J, Vassen L (2018). GFI1 is required for RUNX1/ETO positive acute myeloid leukemia. Haematologica..

[122] Mulloy JC, Jankovic V, Wunderlich M, Delwel R, Cammenga J, Krejci O (2005). AML1-ETO fusion protein up-regulates TRKA mRNA expression in human CD34+ cells, allowing nerve growth factor-induced expansion. Proc Natl Acad Sci USA..

[123] Shimada H, Ichikawa H, Nakamura S, Katsu R, Iwasa M, Kitabayashi I (2000). Analysis of genes under the downstream control of the t(8;21) fusion protein AML1-MTG8: overexpression of the TIS11b (ERF-1, cMG1) gene induces myeloid cell proliferation in response to G-CSF. Blood..

[124] Tiacci E, Pileri S, Orleth A, Pacini R, Tabarrini A, Frenguelli F (2004). PAX5 expression in acute leukemias: higher B-lineage specificity than CD79a and selective association with t(8;21)-acute myelogenous leukemia. Cancer Res..

[125] Fortier JM, Payton JE, Cahan P, Ley TJ, Walter MJ, Graubert TA (2010). POU4F1 is associated with t(8;21) acute myeloid leukemia and contributes directly to its unique transcriptional signature. Leukemia..

[126] Dunne J, Mannari D, Farzaneh T, Gessner A, van Delft FW, Heidenreich O (2012). AML1/ETO and POU4F1 synergy drives B-lymphoid gene expression typical of t(8;21) acute myeloid leukemia. Leukemia..

[127] Ponnusamy K, Chen-Wichmann L, Kuvardina ON, Lausen J, Henschler R, Wichmann C (2014). The truncated RUNX1/ETO activates VLA-4-dependent adhesion and migration of hematopoietic progenitor cells. Haematologica..

[128] Bristow CA, Shore P (2003). Transcriptional regulation of the human MIP-1alpha promoter by RUNX1 and MOZ. Nucleic Acids Res.

[129] Stoner SA, Liu KTH, Andrews ET, Liu M, Arimoto KI, Yan M (2020). The RUNX1-ETO target gene RASSF2 suppresses t(8;21) AML development and regulates Rac GTPase signaling. Blood Cancer J..

[130] Claus R, Fliegauf M, Stock M, Duque JA, Kolanczyk M, Lübbert M (2006). Inhibitors of DNA methylation and histone deacetylation independently relieve AML1/ETO-mediated lysozyme repression. J Leukoc Biol..

[131] Liddiard K, Hills R, Burnett AK, Darley RL, Tonks A (2010). OGG1 is a novel prognostic indicator in acute myeloid leukaemia. Oncogene..

[132] Ponnusamy K, Kohrs N, Ptasinska A, Assi SA, Herold T, Hiddemann W (2015). RUNX1/ETO blocks selectin-mediated adhesion via epigenetic silencing of PSGL-1. Oncogenesis..

[133] Yang G, Khalaf W, van de Locht L, Jansen JH, Gao M, Thompson MA (2005). Transcriptional repression of the Neurofibromatosis-1 tumor suppressor by the t(8;21) fusion protein. Mol Cell Biol.

[134] Kohrs N, Kolodziej S, Kuvardina ON, Herglotz J, Yillah J, Herkt S (2016). MiR144/451 Expression Is Repressed by RUNX1 During Megakaryopoiesis and Disturbed by RUNX1/ETO. PLoS Genet.

[135] Vangala RK, Heiss-Neumann MS, Rangatia JS, Singh SM, Schoch C, Tenen DG (2003). The myeloid master regulator transcription factor PU.1 is inactivated by AML1-ETO in t(8;21) myeloid leukemia. Blood..

[136] Zhuang WY, Cen JN, Zhao Y, Chen ZX. Epigenetic silencing of Bcl-2, CEBPA and p14(ARF) by the AML1-ETO oncoprotein contributing to growth arrest and differentiation block in the U937 cell line. Oncol Rep. 2013;30:185–92. 10.3892/or.2013.2459 23673926

